# A Comparative Study of the Triglycerides/HDL Ratio and Pseudocholinesterase Levels in Patients with Bladder Cancer

**DOI:** 10.3390/diagnostics12020431

**Published:** 2022-02-07

**Authors:** Felice Crocetto, Savio Domenico Pandolfo, Achille Aveta, Raffaele Martino, Francesco Trama, Vincenzo Francesco Caputo, Biagio Barone, Marco Abate, Enrico Sicignano, Simone Cilio, Gianluca Russo, Matteo Massanova, Concetta Di Vito, Ciro Imbimbo, Giovanni Tarantino

**Affiliations:** 1 Department of Neuroscience, Reproductive Sciences and Dentistry, Federico II University, 80131 Naples, Italy; felice.crocetto@unina.it (F.C.); pandolfosavio@gmail.com (S.D.P.); achille-aveta@hotmail.it (A.A.); raffaele.martino88@yahoo.it (R.M.); vincitor@me.com (V.F.C.); biagio.barone@unina.it (B.B.); marcoabate5@gmail.com (M.A.); enrisici90@gmail.com (E.S.); simocilio.av@gmail.com (S.C.); tina.dvt@tiscali.it (C.D.V.); ciro.imbimbo@unina.it (C.I.); 2 Division of Urology, VCU Health, Richmond, VA 23219, USA; 3 Andrology and UroGynecology Clinic, AOSP Santa Maria di Terni, 05100 Terni, Italy; francescotrama@gmail.com; 4 Department of Public Health, University of Naples Federico II, 80138 Naples, Italy; gianlucar93@libero.it; 5 Department of Urology, Queen Elizabeth, University Hospital, Glasgow G51 4TF, UK; matteo.massanova@gmail.com; 6 Department of Clinical Medicine and Surgery, Federico II Medical School of Naples, 80138 Naples, Italy

**Keywords:** bladder cancer, TG/HDL ratio, pseudocholinesterase

## Abstract

Background: Lipid alterations may serve as potential tumour biomarkers. The ratio of triglycerides to HDL cholesterol (TG/HDL ratio) is associated with various cancers. Pseudocholinesterase (PChE) activity, involved in TG hydrolysis, plays an important role in the metabolism of lipoprotein. There is scarce data assessing the reliability of both the TG/HDL ratio and PChE levels in correctly classifying patients suffering from bladder cancer. Methods: Three hundred and ninety-six patients undergoing cystoscopy or transurethral resection of the bladder (TURB), broken into two major groups, i.e., patients with histologically confirmed, non-metastatic bladder cancer (*n* = 208) and without bladder cancer (no bladder cancer, *n* = 188), formed the study population. The last group was split into two subgroups consisting of a cohort of patients never suffering from bladder cancer but with other bladder diseases (no CaBD, *n* = 100) and another cohort formed by patients characterised by eradicated bladder cancer after TURB with no recurrence during a three-month follow-up (previous bladder cancer, *n* = 88). Pieces of information by both metabolic derangement (the presence of type 2 diabetes mellitus), hypertension and lipid profile were retrieved from patient records upon entry to the study. Sensitivity, specificity, areas under the ROC (AUROC) of the TG/HDL ratio, and PChE levels were used in diagnostic decision making. Results: The TG/HDL ratio as well as PChE concentrations of bladder cancer patients were significantly different when compared to those with previous bladder cancer and the no CaBD patients (*p* = 0.023 and 0.0004, respectively). There was an independent role of both the TG/HDL ratio and PChE levels in predicting the presence of bladder cancer (OR: 1.22 and 0.99, respectively), but the reliability of the TG/HDL ratio (AUROC: 0.587) was superior to that of PChE levels (AUROC: 0.374). The AUROC of a new parameter resulting from the combination of the TG/HDL ratio with PChE levels showed a further increment in the discriminant power of the bladder cancer presence (0.6298), interestingly with a negative predictive value (89%) according to the Bayesian approach. The cut-off of the TG/HDL ratio, the main marker of the present study that better distinguishes bladder cancer from no bladder cancer patients, was 2.147. Discussion and Conclusions: The reliability of the TG/HDL ratio is based on the fact that this parameter likely mirrors the insulin resistance (IR) underlying bladder cancer patients. Furthermore, PChE levels evidence both IR and the associated non-alcoholic fatty liver disease. The TG/HDL ratio and PChE levels as well as their combined use could help physicians to assess/confirm the presence of this very common cancer, where early detection is important to ensure the best therapeutical approach.

## 1. Introduction

Bladder cancer (BCa) is very common in men but less prevalent in women, occurring mainly in old people, with an age-standardised rate of 10^5^ of 15.9, https://www.wcrf.org/dietandcancer/bladder-cancer-statistics/ (accessed on 18 January 2022).

As well as the most frequent risk factor, which is cigarette smoking, metabolic factors could also play a role. On the basis of recent data, the pathological stage of BCa was remarkably related to the criteria of metabolic syndrome (MS) in a retrospective study of 169 patients. Specifically, MS was significantly associated with a high histological grade. Low high-density lipoprotein (HDL) was related to the T stage, but no other components of MS were associated with a high stage or grade [1]. Accordingly, a molecular classification has been established for BCa based on the expression of metabolism-related genes [2].

Indeed, altered lipid metabolism is one of the most prominent metabolic disorders in any cancer. In fact, enhanced synthesis or uptake of lipids contributes to cancer cell growth and tumour formation [3]. Consequently, there is a large consensus on accepting that lipid alterations may serve as potential tumour biomarkers [4]. 

Trying to deepen the mechanisms, several pieces of research have demonstrated that lipid metabolism is reprogrammed in cancers. Specifically, fatty acid oxidation, lipolysis as well as lipophagy, extracellular lipid uptake, the cholesterol genesis, and more importantly de novo lipogenesis, are activated [5].

Accordingly, the latter pathway (converting excess carbohydrates into fatty acids, successively esterified to store triglycerides) forms the metabolic phenotype of BCa [6]. For this reason, the interest in investigating the serum lipid profile in this specific cancer is growing.

The ratio of triglycerides to HDL-cholesterol (TG/HDL ratio), initially proposed by Gaziano et al. as an atherogenic index [7], is an independent predictor of poor clinical outcomes in triple-negative breast cancer patients [8]. Furthermore, the TG/HDL ratio was positively associated with clinical features of endometrial cancer, such as tumour stage and pathogenetic type [9]. The TG/HDL ratio is an efficient and independent prognostic factor to predict the five-year case fatality of gastric cancer patients and to improve the efficacy of its prognostic nomogram [10].

On the other hand, the inverse correlation between TG concentration and both pseudocholinesterase (PChE) activity and lipoprotein lipase activity (LPL) suggests that PChE, similarly to LPL, may be involved in TG hydrolysis [11], in the sense that both LPL and PChE play important roles in the metabolism of lipoproteins and participate in lipoprotein interconversions. Thus, authors have long since studied PChE as a tumour marker [12]. In fact, there is an interesting investigation showing low PChE levels in various malignancies of the head and neck as well as of the uterine cervix [13]. Decreased pre-treatment serum PChE concentration is an independent predictor for higher pT stage, shorter overall survival, and cancer-specific survival in patients with upper tract urothelial carcinoma [14]. Low serum levels of PChE at recurrence of pancreatic cancer are a poor prognostic factor and relate to systemic disorder and nerve plexus invasion (a sign of likely cancer cachexia) [15]. Furthermore, recent results suggest that PChE analysis before chemotherapy is a promising prognostic marker for advanced gastric cancer [16]. Finally, serum levels of PChE were significantly lower in patients with muscle-invasive BCa than in those with non-muscle-invasive BCa, or in patients with high- compared to low-grade cancer [17]. In everyday practice, PChE remains an index of impaired liver function, due to its decreased synthesis [18].

Although a number of biochemical parameters have been associated with specific cancers, researchers have achieved only partial success in proposing them as diagnostic tools.

Since the recurrence and mortality rates of BCa are high, suitable biomarkers for early detection are needed [19].

Specifically, to the best of our knowledge, there is scarce data assessing the reliability of both the TG/HDL ratio and PChE levels in correctly classifying patients suffering from BCa. We aimed at filling this gap in light of dyslipidaemia being a distinctive figure of BCa.

## 2. Methods

### 2.1. Study Design and Data Source

We retrospectively evaluated the clinical and laboratory data retrieved from administrative records of 396 patients. These patients were admitted to a tertiary University/Hospital, from January 2018 to December 2020, for asymptomatic or gross haematuria or irritative voiding symptoms.

They underwent renal function, urinary cytology, and ultrasonography as a first diagnostic step. Successively, there was an evaluation with cystoscopy to correctly diagnose the nature of suspicious lesions. Transurethral resection of the bladder (TURB) allowed for definitive diagnosis, staging, and primary treatment of the tumour. Computed tomography scan or magnetic resonance imaging were used to find out whether the BCa had spread.

Accordingly, the whole population under analysis was broken in two major groups, i.e., patients with histologically confirmed, non-metastatic BCa (*n* = 208) and those without BCa (*n* = 188). 

This last group was further split into two subgroups, consisting of a cohort of patients never suffering from BCa but with benign bladder diseases (no CaBD, *n* = 100) and another cohort formed by patients characterised by eradicated BCa after TURB with no recurrence during a three-month follow-up (previous BCa, *n* = 88). 

Patients whose records did not contain sufficient data were excluded from the analysis.

This article does not report on primary research, and subsequently analysed data were gathered during the hospital stay. Our analysis scrutinised the data of these cohorts, respecting complete anonymity, and was performed internally as part of an evaluation to improve our quality of care. Patients were diagnosed and treated according to the European guidelines and agreements [20]. Testing blood as well as recording all other variables included in our analysis was essential for confirming the diagnosis and classifying patients. It was performed for each patient without unsuccessful results in the course of examination and as part of routine care and was in no way an add-on for the purposes of the research. For these reasons, no ethical approval was requested, and informed written consent was not obtained from each subject.

### 2.2. Bladder Cancer Pathology

The stage and the grade of BCa were determined by examining the tissue sample, removed during a TURB procedure from the area where cancer may have existed, according to recent well-accepted guidelines [21].

### 2.3. Metabolic Assessment at Entry

The TG/HDL ratio was calculated by dividing the serum levels of these two lipids. In a nutshell, the Tg/HDL ratio is considered ideal when it is two or less, four is considered high, and six or greater is considered too high [22].

Patients were diagnosed as suffering from type 2 diabetes mellitus (T2DM) if their fasting blood glucose was 126 mg/dL (7 mmol/L) or higher on two separate tests, or if they were on anti-diabetic drugs or undergoing treatment with insulin.

### 2.4. Diagnosis of Systemic Arterial Hypertension

Data for systolic/diastolic blood pressure (SBP/DBP) were the average of three consecutive detections taken after having allowed the subjects to rest for five minutes in the sitting position. Patients on current anti-hypertensive drugs were considered as suffering from hypertension, even if it was controlled.

### 2.5. Laboratory Data

The lipids profile confirmed serum levels of TG (n.v. < 150 mg/dL), total cholesterol (TC, n.v. < 200 mg/dL), HDL (n.v. > 40 mg/dL for men and >50 mg/dL for women), and low-density lipoprotein (LDL, n.v. < 100 mg/dL). In the liver function tests, aspartate aminotransferase (AST, n.v. < 34 UI), alanine aminotransferase (ALT, n.v. < 55 UI), gamma-glutamic transpeptidase (GGT, n.v. 12–64 UI), alkaline phosphatase (ALP, n.v. 40–150 UI), and lactate dehydrogenase (LDH, n.v. 125–243 UI) were analysed. All the previous laboratory parameters, as well as serum uric acid (n.v. 3.5–7.2 mg/mL), serum creatinine (n.v. 0.72–1.25 mg/dL), and platelets (n.v. 130–400^3^/uL), were measured/counted according to in-house procedures. 

### 2.6. Statistics

To run correct statistical tests and thus draw acceptable conclusions, we used a double approach: data were analysed after splitting the whole examined population into two groups, i.e., no BCa and Bca, and then dividing the no BCa group into previous BCa and no CaBD subgroups to ultimately form three well-defined cohorts. 

Variables that were not normally distributed according to the Shapiro–Wilk test analysis were expressed as the median plus interquartile range (IQR), while those derived from a normally distributed population were reported as the mean plus standard deviation (SD). 

Differences between medians of two groups were detected by a two-sample Wilcoxon rank-sum (Mann–Whitney) test. When comparing variables not normally distributed in more than two groups, the ANOVA Kruskal–Wallis (K-W) test with post hoc analysis using the Dunn test was evaluated. The extended Mantel–Haenszel with ANOVA (transformation in ranks) analysis (https://www.statology.org/friedman-test-stata/, accessed on 18 January 2022) was used when adjusting for age. This tool is the non-parametric alternative of the parametric repeated measures ANOVA, and it is known as the Friedman test.

The two-way table with measures of association, and the related Pearson’s chi-squared test, were used to weigh frequencies when dealing with categorical variables.

An ordered probit model, which is a particular method of regression analysis used for prediction, was applied to estimate relationships between an ordinal dependent variable, i.e., staging, and independent variables, i.e., the TG/HDL ratio and PChE levels, evaluating the coefficient with its standard error (Std. err.), the z-value, *p* >|z|, and the 95% confidence interval (CI). Pseudo R-square, as a statistical measure of whether the model better predicts the outcome, was also reported.

The logistic regression was carried out to predict the presence/absence of BCa by using both the TG/HDL ratio and PChE levels as independent variables after merging the no CaBD and previous BCa groups into a unique variable, no BCa. Odds ratio, Std. err., the z-value, *p* > |z|, and 95% CI were shown.

The ROC analysis (DeLong method) was used in diagnostic decision making of the BCa presence/absence using the selected laboratory parameters, after merging no CaBD and previous BCa patients into a single group, i.e., the no BCa group. To measure the performance of the binary classification test (index test), the area under the receiver operating characteristic curve (AUROC/AUC) was evaluated to identify the most appropriate models (the highest specificity and sensitivity) under the nonparametric assumption, i.e., distribution free. As a post-estimation, the sensitivity/specificity versus probability cut-off plot indicated where the intersection point between sensitivity and specificity lies, in order to show the accuracy of the index test. As is common in the literature, we chose a 50% cut-off to predict the positive and negative values. This cut-off is useful for trying to balance the harms of the type I and type II errors. 

The best cut-off, coupled with the sensitivity, specificity, positive likelihood ratio (LR+), and the negative likelihood hood (LR–), was studied, pointing out that the higher the LR+ is than one, the more likely the outcome. On the contrary, the lower the LR– is than one, the less likely the outcome.

The cut-off with the highest specificity and sensitivity was calculated by means of the Youden Index according to [23]. The test threshold is the probability of disease below or above which there would be no further testing that would be considered necessary for that circumstance.

The test equality of more ROC areas was performed to compare the diagnostic performance of several variables.

In order to derive the posterior distribution of the probability of the BCa event, the Bayesian logistic regression—Markov chain Monte Carlo—was used by adopting the Random-Walk Metropolis–Hastings sampling. According to this tool, the positive predictive value (PV+) was generated, as well as the negative predictive value (PV−). Diagnostic tests need to be considered in context. As the prevalence of a disease increases, so does the positive predictive value. 

The power analysis to establish the minimum sample size was performed by calculating the difference in means and SDs (two-sided) of two groups (no BCa and BCa patients).

A *p*-value < 0.05 was accepted as significant.

Statistical analyses were run on Stata 17.0., Stata Corp., 4905 Lakeway Drive College Station, TX 77845, USA.

## 3. Results

The main demographic, clinical, and laboratory characteristics of patients belonging to the three groups forming the whole population are shown in Table 1. The staging of BCa and previous BCa patients as well as the clinical diagnosis of no CaBD are evident in Supplementary Tables S1–S3.

### 3.1. Prevalence

BCa patients were older than patients belonging to the previous BCa and no CaBD groups and male patients were overly represented in all groups. BMI was similar throughout the whole population. Hypertension was equally represented throughout the three groups.

The BCa group showed higher levels of uric acid, creatinine, and triglycerides and lower concentrations of HDL when compared with the values of other groups.

### 3.2. Behaviour of the Principal Parameters According to Their Numerosity

**Figure 1:**
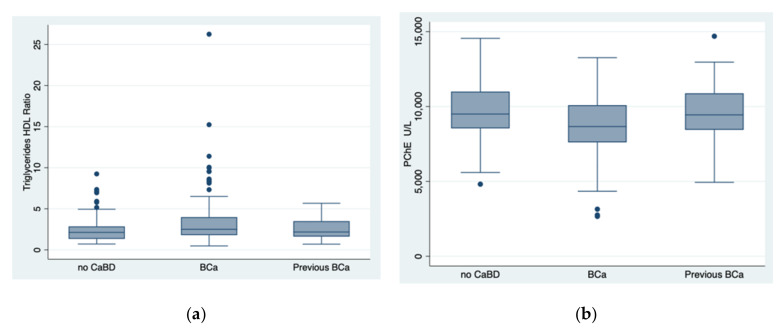
The behaviour of the TG/HDL ratio and pseudocholinesterase levels in the three groups, i.e., no cancer but bladder disease, bladder cancer, and previous bladder cancer. BCa, bladder cancer; no CaBD, no cancer but bladder disease; previous BCa, previous bladder cancer; PChE, pseudocholinesterase. Indeed, there was a statistically significant difference (Kruskal–Wallis test) for the TG/HDL ratio between the groups (*p* = 0.023): specifically, between no CaBD and BCa groups (*p* = 0.005), as well as between previous BCa and BCa groups (*p* = 0.04). (**a**) Similarly, there was a statistically significant difference found by the same test for PChE between the groups (*p* = 0.0005): specifically, between no CaBD and BCa groups (*p* = 0.0004), as well as between previous BCa and BCa groups (*p* = 0.0019) (**b**).

Studying the TG/HDL ratio in the three groups, i.e., BCa (*n* = 162), previous BCa (*n* = 72), and no CaBD (*n* = 71), the K-W test showed that there was a statistically significant difference between the groups (*p* = 0.023): specifically, according to the post hoc analysis, between no CaBD and BC groups (*p* = 0.005), as well as between previous BCa and BCa groups (*p* = 0.04), as shown in Figure 1a. When adjusting the difference of the TG/HDL ratio found in the three groups for age, the significance decreased (*p* = 0.057).

The same statistical test, conducted to determine if the PChE concentration was dissimilar for the three cohorts, i.e., BCa (*n* = 174), previous BCa (*n* = 73), and no CaBD (*n* = 77), highlighted that there was a statistically significant difference between the groups (*p* = 0.0005): according to the post hoc analysis, between no CaBD and BCa groups (*p* = 0.0004), as well as between previous BCa and BCa groups (*p* = 0.0019), as shown in Figure 1b. The result was still significant when the difference in the PChE concentration was adjusted for age, *p* = 0.009.

When analysing whether the creatinine concentration differed among the three groups, i.e., BCa (*n* = 208), previous BCa (*n* = 87), and no CaBD (*n* = 99), there was a statistically significant difference between the groups (*p* = 0.0002): based on the post hoc analysis, between no CaBD and BCa groups (*p* = 0.0000), as well as between BCa and previous BCa groups (*p* = 0.015). The differences were independent from age, *p* = 0.016.

Finally, examining whether the uric acid concentration varied among the three groups, i.e., BCa (*n* = 172), previous BCa (*n* = 73), and no CaBD (*n* = 78), there was a statistically significant difference between the groups (*p* = 0.046): after the post hoc analysis, between no CaBD and BCa groups (*p* = 0.0067). When adjusting for age, the significant difference of uric acid concentration in the previous two groups was lost, *p* = 0.44.

**Figure 2:**
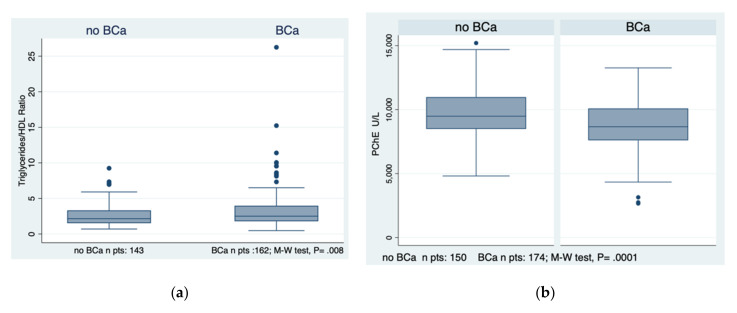
The behaviour of the TG/HDL ratio and pseudocholinesterase levels in the two major groups, i.e., bladder cancer and no bladder cancer is evidenced in subfigures (**a**,**b**). BCa, bladder cancer; no BCa, no bladder cancer; PChE, pseudocholinesterase; M-W test, Mann–Whitney test.

Merging the two groups, i.e., previous BCa and no CaBD, into one group, labelled the no BCa group, the TG/HDL ratio and PChE levels differentiated this new group from the BCa group with a significance of *p* = 0.008 and *p* = 0.0001, respectively, as evident in Figure 2a,b. 

### 3.3. Predictions by the “Index” Parameters

As a powerful model of association, e.g., the logistic regression, the TG/HDL ratio and PChE levels positively and negatively predicted the presence/absence of BCa, as detailed in Table 2.

Studying the association between the staging of BCa and the two diagnostic parameters, i.e., TG/HDL ratio and PChE concentration, a significant prediction was present only for PChE concentration, as shown in Table 3.

### 3.4. Discriminant Analysis of the Presence of Bladder Cancer 

**Figure 3:**
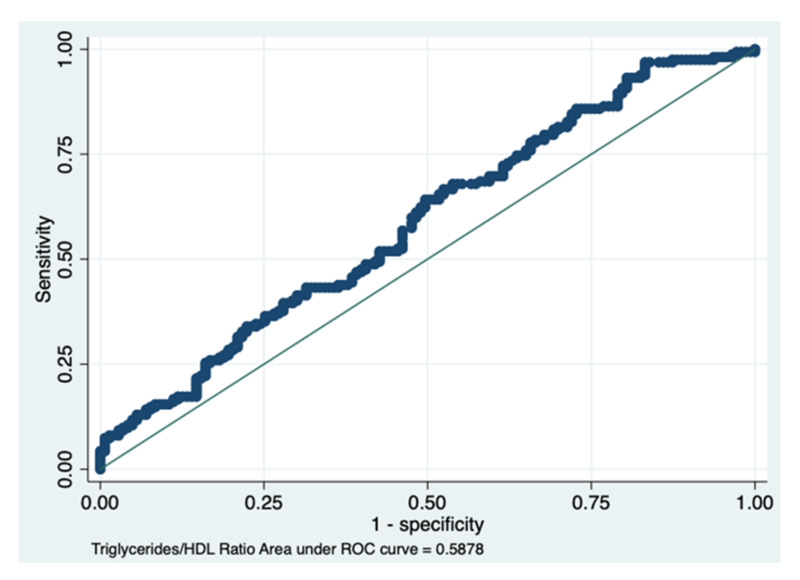
The area under the ROC curve of the triglycerides/HDL ratio. Details are presented in Table 4.

**Figure 4:**
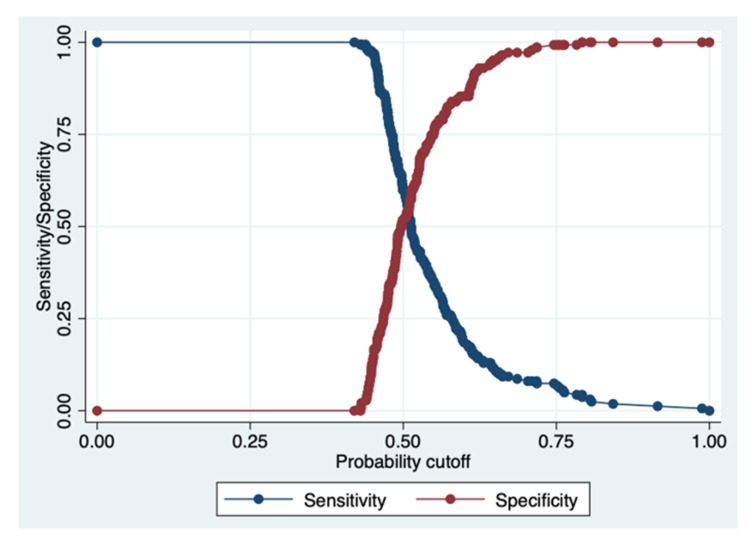
Plot of sensitivity/specificity versus probability cut-off point. This post-estimation, i.e., the sensitivity/specificity versus probability cut-off plot, indicates that at a probability cut-off of 0.50, both the sensitivity and specificity for the triglycerides/HDL ratio are satisfactory. In fact, the two lines (red and blue) merge at a level superior to 50%, indicating a sufficient discrimination power (see details in Table 4).

The AUC of the TG/HDL ratio obtained a value of 0.587, with a 95% CI between 0.523 and 0.651, indicating a sufficient discriminant power (Figure 3 and Figure 4, Table 4).

The Youden Index analysis to assess the best cut-off of the TG/HDL ratio for indicating the presence/absence of BCa obtained the following results, as shown in Table 5.

**Figure 5:**
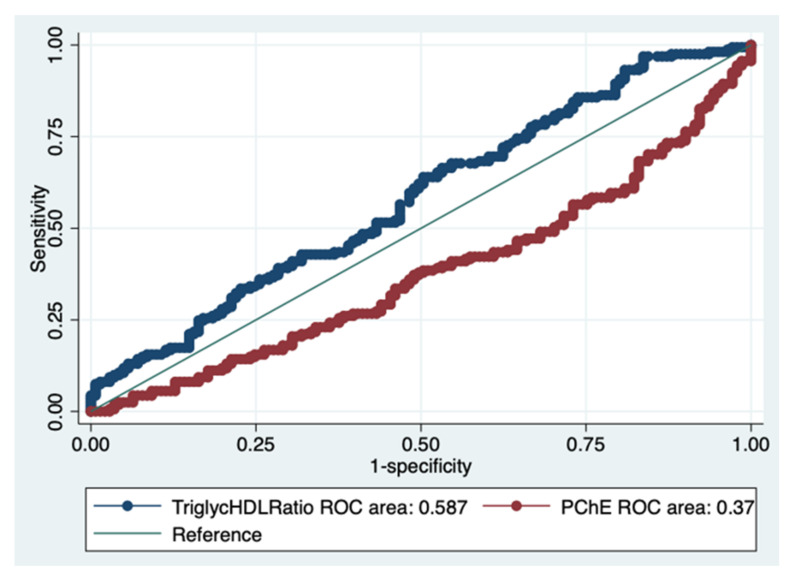
Comparison of the two ROC areas, i.e., the TG/HDL ratio and the pseudocholinesterase levels, as performance evaluation measures. The TG/HDL ratio showed a higher AUCROC than that of the pseudocholinesterase levels.

The AUROC of PChE was 0.374, showing a reduced value compared to that of the TG/HDL ratio (chi^2^ = 22.48, Prob > chi^2^ = 0.0000), presented in Figure 5.

**Figure 6:**
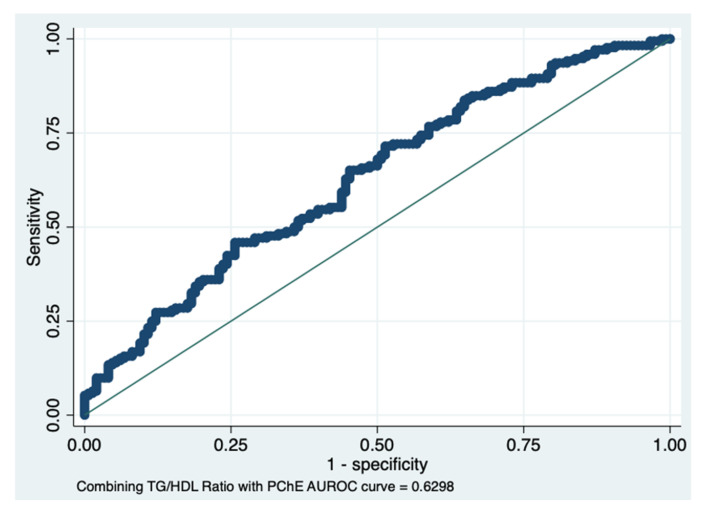
ROC area combining the triglycerides/HDL ratio with pseudocholinesterase levels. A new parameter generated by combining the TG/HDL ratio with pseudocholinesterase levels showed an improvement in the discrimination power of the presence of BCa.

The AUROC result, combining the TG/HDL ratio with PChE levels, i.e., 0.63, showed an improvement in diagnosing the presence of BCa (sensitivity) and a slight decrease in specificity (false positive rate), as shown in Figure 6. The sensitivity and specificity were 66.22% and 50%, respectively, with 58.75% correctly classified.

In order to take a frequentist approach, the Bayesian logistic regression, used for predicting the presence of BCa by the new parameter obtained by combining the TG/HDL ratio with PChE levels, obtained an OR of 3.33 with a SD of 0.93. 

Considering a BCa prevalence of 15.9% according to [24], the PV+ of the combined test was 0.20, indicating that among the people who test positive, only 20% actually have the disease, while the PV− was 89%, suggesting that for those that test negative, about 90% do not have the disease.

The power analysis established that the minimum sample was less than ten patients per group concerning both the TG/HDL ratio and PChE levels.

## 4. Discussion

The main results of this retrospective study were: (a) The TG/HDL ratio as well as PChE concentrations of BCa patients were significantly different when compared to those of previous BCa and no CaBD patients. (b) There was an independent role of both the TG/HDL ratio and PChE levels in predicting the presence of BCa, but the diagnostic reliability of the TG/HDL ratio was superior to that of PChE. (c) The AUROC of a new parameter resulting from the combination of the TG/HDL ratio with PChE levels showed a further increment in the discriminant power of the presence of BCa, interestingly with a negative predictive value.

Concerning the cut-off of the TG/HDL ratio (one of the two markers of the present study that better distinguishes BCa from no BCa patients), i.e., 2.147, we stress that this value was higher than that proposed by Dai et al. [8], Luo et al. [9], and by Sun et al. [10], but it coincided with that found in an Italian study [25]. Interestingly, a cut-off similar to ours, i.e., 2.27, was used in a large multi-ethnic cohort of obese patients to identify subjects with insulin resistance (IR) [26]. Another piece of proof linking the TG/HDL ratio to IR was found when investigating NAFLD and carotid artery intima-media thickness [27]. Indeed, the selected populations of the previously quoted studies were formed by young or very young subjects, differently from our cohorts. Dealing with adult patients, a TG/HDL ratio cut-off of 1.11 was used to discriminate those with IR [28]. 

These observations bring us to a possible interpretation of what mechanisms are at the basis of the TG/HDL ratio in BCa patients. It has long been known that high levels of TG and low levels of HDL are related to IR [29], but only recently has it been found that this dysmetabolic status is associated with BCa [30]. Thus, the accuracy of the TG/HDL ratio relies on the fact that this parameter likely mirrors the IR underlying BCa patients. The importance of IR in BCa patients is testified by a recent meta-analysis based on ten case-control and fourteen cohort studies that showed a positive association between T2DM and risk of BCa [31]. In the same way, blood pressure levels, strictly linked to IR [32], were positively associated with muscle-invasive BCa but not with non-muscle-invasive BCa in a Swedish cohort [33].

Comparing the results of our study with other recent findings, it is noted that the decreased PChE levels found in our cohort overlapped those observed in recent research on BCa, showing lower concentrations in patients with high-grade cancer [17]. In agreement with our results, findings from another clinical investigation showed that decreased PChE values were associated with shorter recurrence-free survival in patients with non-muscle-invasive BCa undergoing TURB [34].

In an attempt to explain the mechanisms underlying the decreased levels of PChE in BCa patients, recent data showed that PChE concentrations are independently associated with the levels of HbA1c [35] (a simple and reliable marker of IR [36]), contributing to the development of non-alcoholic fatty liver disease (NAFLD) even at normal range values [37]. Indeed, we should state that lipolysis (a pathway which is likely involved in PChE) and β-oxidation are intimately linked, playing an essential role in the pathogenesis of NAFLD. These observations lend credence to the fact that PChE levels also mirror the IR status and the presence of NAFLD in BCa patients, confirming the findings of a previous study [29].

Last but not least, these two laboratory parameters, i.e., the TG/HDL ratio and PChE levels, are easily available with a favourable cost–benefit analysis.

## 5. Limitations 

Although in this discovery cohort the minimum sample size was sufficiently represented, our findings should be confirmed in a validation cohort and in different populations before claiming their utility in helping to diagnose the presence/absence of BCa. Moreover, further research addressing mechanisms underlying these laboratory findings is needed to better understand the connection between lipid dysmetabolism and cancerogenesis. Similarly, the role of hypertension, often associated with important metabolic abnormalities, including those concerning lipid metabolism [38], should be further studied.

## 6. Conclusions

There is an essential demand for non-invasive, sensitive, fast, and inexpensive biomarkers to ensure an optimal clinical management of BCa patients. This retrospective study showed that simple tests, i.e., the TG/HDL ratio and PChE levels, as well as their combined use, could help physicians to assess/confirm the presence of this very common cancer, where early detection is important to ensure the best therapeutical approach.

## 7. Future Directions

A desirable target should be an early diagnosis of BCa before it has spread into the bladder wall. Unfortunately, the natural history of BCa is one of recurrence and progression, with recurrence being most common in the first 12–24 months [39]. Consequently, urologists are forced to treat BCa by radical cystectomy [40]. Thus, adopting some biomarkers to monitor the progression of residual disease or recurrence of invasive cancer could be a good option to consider. In this context, liquid biopsy, a method that has been widely investigated over the last decade [41], and neutrophil percentage-to-albumin ratio, which predicts mortality in bladder cancer patients [42], could be good tools to ascertain the reliability of the laboratory indices proposed, i.e., the TG/HDL ratio and PChE levels.

Finally, some other parameters, reflecting altered lipid metabolism, could be evaluated, such as: atherogenic index of plasma, calculated as (lg(TG/HDL)) [43], non-HDL, calculated as TC – HDL [44], LDL/HDL ratio [45], atherogenic index, calculated as non-HDL/HDL [46], and lipoprotein combined index, calculated as TC * TG * LDL/HDL ratio [47]. 

## Figures and Tables

**Table 1:** Characteristics of patients.

	BCa	Previous BCa	No CaBD	*p*-Value
**Age (years),** **median (IQR)**	**71 (57–73)**	**69.5 (64–75)**	**66.5 (57–73)**	0.0007 *
**Gender (M/F), *n***	**177/31**	**71/17**	**67/33**	0.001 **
BMI	26.6 (24.1–29.4)	27 (24.7–29.2)	26.7 (24.1–29.4)	0.65 *
**Triglycerides/HDL Ratio**	**2.5 (1.8–4)**	**2.1 (1.6–3.5)**	**2.1 (1.3–2.8)**	0.023 *
**PChE (U/L)**	**8660 (7605–10,088)**	**9440 (8440–10,979)**	**9497 (8538–10,993)**	0.0005 *
Glucose (mg/dL)	97 (87–115)	97 (85–109)	91.5 (81–108)	0.15 *
Total Cholesterol (mg/dL)	172.5 (148–203)	175.5 (152–216)	185 (160–209)	0.12 *
LDL Cholesterol (mg/dL)	107 (81–134)	115.5 (90–150)	116 (93–138)	0.11 *
**HDL Cholesterol** **(mg/dL)**	**42 (36–49)**	**47 (37.5–56)**	**46 (40–55)**	0.0065 *
**Triglycerides** **(mg/dL)**	**112 (84–144)**	**106.5 (77–140)**	**97.5 (75–127.5)**	0.03 *
**Uric Acid** **(mg/dL)**	**5.8 (4.7–6.8)**	**5.5 (4.6–6.8)**	**5.3 (4.4–5–9)**	0.046 *
Ferritin (ng/mL)	89 (43–176)	71.5 (43.5–114)	83 (35.5–136.5)	0.28 *
**Creatinine** **(mg/dL)**	**1 (0.8–1.2)**	**0.9 (0.8–1–1)**	**0.8 (0.7–1)**	0.0002 *
T2DM, *n*	50	15	15	0.127 **
Hypertension, *n*	131	54	51	0.124 **
GGT (U/L	20 (15–32)	19 (16–29)	22 (15–30)	0.8 *
ALT (U/L)	18 (13–22)	17 (12–23)	18 (13–22)	0.55 *
AST (U/L)	19 (15–22)	19 (17–23)	18 (15–22)	0.34 *
ALP (U/L)	81 (67–99)	74 (63–87)	80 (66–101)	0.16 *
**LDH (U/L)**	**186 (165–212)**	**193 (172–222)**	**180 (158–208)**	0.01 *
Platelets (10^3^/uL)	205 (168.5–246.5)	213 (178.5–230)	216 (178–266)	0.16 *

* Kruskal–Wallis H test. ** Pearson’s chi-squared. *n*, number; BCa, bladder cancer; no CaBD, no cancer but bladder disease; GGT, gamma-glutamyl transferase; ALT, alanine aminotransferase; AST, aspartate aminotransferase; LDH, lactate dehydrogenase; PChE, pseudocholinesterase; ALP, alkaline phosphatase; T2DM, type 2 diabetes mellitus.

**Table 2:** Prediction of the presence of bladder cancer by the triglycerides/HDL ratio and pseudocholinesterase levels. Logistic regression: number of observations = 302, Pseudo R^2^ = 0.0619.

d.v.	BCa (Yes/No)	Odds Ratio	Std. Err.	z	*p* >|z|	95% CI
**i.v.**	Triglycerides/HDL Ratio	1.22022	0.0848785	**2.86**	**0.004**	1.064706–1.398455
**i.v**	PChE	0.9997446	0.0000648	**−** **3.94**	**0.0000**	0.9996176–9998715

BCa, bladder cancer; PChE, pseudocholinesterase; d.v., dependent variable; i.v., independent variable. The 95% confidence intervals of the triglycerides/HDL ratio lie in a larger range than those of PChE. With a note of caution (low pseudo R^2^) in their interpretation, the TG/HDL ratio did not predict the severity (stage) of bladder cancer in the BCa group, differently from PChE levels, which negatively predicted it (Table 3).

**Table 3:** Prediction of the staging of bladder cancer by the triglycerides/HDL ratio and pseudocholinesterase levels.

**Ordered Probit Regression: Number of Observations = 162, Pseudo R^2^ = 0.0011**						
**d.v.**	**Staging**	**Coefficient**	**Std. Err.**	**Z**	***p* > |z|**	**95% CI**
i.v.	Triglycerides/HDL Ratio	−0.02272	0.03165	−0.72	0.47	0.08476–0.03930
**Ordered Probit Regression: Number of Observations = 174, Pseudo R^2^ = 0.0274**						
**d.v.**	**Staging**	**Coefficient**	**Std. Err.**	**z**	***p*** **> |z|**	**95% CI**
i.v.	PChE	−0.00015	0.00004	**−** **3.67**	**0.000**	−0.00023–0.00007

PChE, pseudocholinesterase. Note: the low pseudo R^2^ of the ordered probit regression concerning the triglyceride/HDL ratio indicates that the model has some limits in predicting the outcome.

**Table 4:** Statistical details concerning the performance of the triglycerides/HDL ratio in ascertaining the presence of bladder cancer.

Area under the ROC Curve of the TG/HDL Ratio	0.5878
True	
Classified D no D	Total
+ 97 68	165
− 65 75	140
Total 162 143	305
Sensitivity Pr (+ D)	**59.8%**
Specificity Pr (− no D)	**52.4%**
Positive predictive value Pr (D+)	58.7%
Negative predictive value Pr (no D)	53.5%
False positive rate for true no D Pr (+ no D)	47.5%
False negative rate for true D Pr (− D)	40.1%
False positive rate for classified + Pr (no D+)	41.2%
False negative rate for classified − Pr (D−)	46.4%
Correctly classified	**56.3%**

Statistical output of the probit model with the post-estimation command >estat class< showing an overall rate of correct diagnosis of 56.3%. Specifically, 52.4% of the no BCa group were correctly classified and 59.8% of the BCa group were correctly classified, as evident in bold. D = disease present; no D = no disease present; Pr = probability; + = positive; − = negative.

**Table 5:** The cut-off with the highest specificity and sensitivity calculated by the Youden Index.

Cut-Off	Sensitivity	Specificity	Correctly Classified	LR+	LR−
<=2.147	64.20%	49.65%	**57.38%**	**0.12752**	**0.72**

## Data Availability

Data are available upon request to the corresponding author.

## Supplementary Materials

The following supporting information can be downloaded at: https://www.mdpi.com/article/10.3390/diagnostics12020431/s1. Table S1: TNM staging system of the enrolled patients suffering from bladder cancer. Table S2: TNM staging system of the enrolled patients suffering from previous bladder cancer. Table S3: Clinical diagnosis of the enrolled patients with other bladder diseases but cancer.
