# Supplementary material for: A Comparative Study of the Triglycerides/HDL Ratio and Pseudocholinesterase Levels in Patients with Bladder Cancer
Source: Diagnostics (Basel). 2022 Feb 7;12(2):431. doi: 10.3390/diagnostics12020431 (PMC8871224; doi:10.3390/diagnostics12020431)
Supplement: Supplementary file 1 [file diagnostics-12-00431-s001.zip › diagnostics-1569469-SI.pdf]

## Supplementary Materials

**Table S1.** TNM staging system of the enrolled patients suffering from bladder cancer.

| Staging of Bladder Cancer Group = 208 | | Number of Patients |
|---|---|---|
| 0a | pTa, Nx, Mx | 77 |
| 0is | pTis, Nx, Mx | 5 |
| | pTis, N0, Mx | 2 |
| I | pT1, Nx, Mx | 60 |
| | pT1, N0, Mx | 10 |
| II | pT2a, Nx, Mx | 7 |
| | pT2a, N0, Mx | 3 |
| | pT2b, Nx, Mx | 11 |
| | pT2b, N0, Mx | 6 |
| IIIa | pT1, N1, Mx | 1 |
| | pT3a, Nx, Mx | 3 |
| | pT3a, N0, Mx | 4 |
| | pT3a, N1, Mx | 3 |
| | pT3b, Nx, Mx | 1 |
| | pT4a, Nx, Mx | 1 |
| | pT4a, N0, Mx | 5 |
| | pT4a, N1, Mx | 3 |
| IIIb | pT1, N2, Mx | 1 |
| | pT3a, N2, Mx | 2 |
| | pT4a, N2, Mx | 3 |

**Table S2.** TNM staging system of the enrolled patients suffering from previous bladder cancer.

| Staging of the Previous Bladder Cancer Group = 88 | | Number of Patients |
|---|---|---|
| 0a | pTa, Nx, Mx | 24 |
| | pTa, N0, Mx | 1 |
| 0is | pTis, Nx, Mx | 1 |
| I | pT1, Nx, Mx | 10 |

| | | |
|---|---|---|
| II | pT2a, Nx, Mx | 4 |
| | pT2a, N0, Mx | 1 |
| III | pT3a, Nx, Mx | 1 |
| | pT3a, N0, Mx | 2 |
| | N/A | 48 |

**Table S3.** Clinical diagnosis of the enrolled patients with other bladder diseases but cancer.

| Clinical Diagnosis—No Cancer Bladder Diseases Group (n=100) | Number of Patients |
|---|---|
| Haematuria | 79 |
| LUTS | 8 |
| Urinary Cytology | 1 |
| N/A | 12 |
| Instrumental analysis—Haematuria- | 79 |
| DH Cystoscopy | 4 |
| Imaging (RMN/TC/US) | 1/35/15 |
| N/A | 24 |
| Surgical procedure | 100 |
| Cystoscopy | 36 |
| TURB | 64 |
